# A Multi-Strategy Visual SLAM System for Motion Blur Handling in Indoor Dynamic Environments

**DOI:** 10.3390/s25061696

**Published:** 2025-03-09

**Authors:** Shuo Huai, Long Cao, Yang Zhou, Zhiyang Guo, Jingyao Gai

**Affiliations:** School of Mechanical Engineering, Guangxi University, Nanning 530004, China; 2211301023@st.gxu.edu.cn (S.H.);

**Keywords:** dynamic environment, visual SLAM, motion blur

## Abstract

Typical SLAM systems adhere to the assumption of environment rigidity, which limits their functionality when deployed in the dynamic indoor environments commonly encountered by household robots. Prevailing methods address this issue by employing semantic information for the identification and processing of dynamic objects in scenes. However, extracting reliable semantic information remains challenging due to the presence of motion blur. In this paper, a novel visual SLAM algorithm is proposed in which various approaches are integrated to obtain more reliable semantic information, consequently reducing the impact of motion blur on visual SLAM systems. Specifically, to accurately distinguish moving objects and static objects, we introduce a missed segmentation compensation mechanism into our SLAM system for predicting and restoring semantic information, and depth and semantic information is then leveraged to generate masks of dynamic objects. Additionally, to refine keypoint filtering, a probability-based algorithm for dynamic feature detection and elimination is incorporated into our SLAM system. Evaluation experiments using the TUM and Bonn RGB-D datasets demonstrated that our SLAM system achieves lower absolute trajectory error (ATE) than existing systems in different dynamic indoor environments, particularly those with large view angle variations. Our system can be applied to enhance the autonomous navigation and scene understanding capabilities of domestic robots.

## 1. Introduction

Numerous home-oriented and human-centered robotic applications have emerged during the past two decades [1,2]. The International Federation of Robotics estimates that sales of household robots reached 40 million units in 2023, corresponding to an annual increase of 25%. For household robots, simultaneous localization and mapping (SLAM) is an essential technology for achieving intelligence and automation [3,4,5]. Visual SLAM, which employs cameras to estimate the robot’s pose and map its surroundings, has been widely studied and received considerable attention in recent years [5].

Nevertheless, operating mobile robots in indoor environments characterized by dynamic human activities remains technically challenging, especially for service applications such as household robotics. This challenge is particularly significant for the advancement of household robotics in real-world applications. A critical limitation arises from the environment rigidity assumption of typical visual SLAM systems, which is invalid in dynamic scenarios [3]. Following this assumption leads to incorrect data associations and thus poor performance of these visual SLAM systems. Therefore, there is an urgent need to resolve the limitations of traditional visual SLAM in dynamic indoor environments, which arises from this assumption [6].

**Figure 1:**
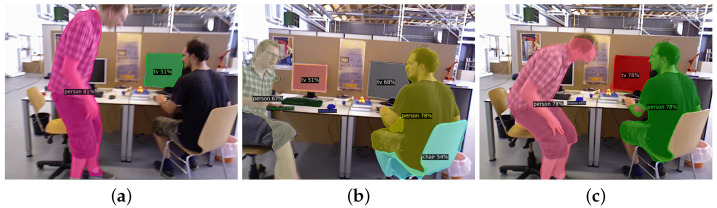
Examples of motion blur impacting semantic information extraction: (**a**) failure of object identification by the segmentation network; (**b**) incomplete segmentation results despite correct object identification; (**c**) degradation of segmentation mask accuracy in boundary regions.

Considering the various proposed methods, the predominant research strategy used in developing solutions involves the combination of semantic information and multi-view geometric information [7,8,9]. Within their frameworks, semantic information plays a crucial role in detecting dynamic objects in scenes, with specialized algorithms subsequently implemented to suppress the negative impact of these dynamic objects on state estimation in SLAM systems. However, the problem of motion blur, caused by dynamic objects within the scene as well as the large view angle variation of the robot, makes it severely challenging for segmentation networks to acquire reliable semantic information from complex dynamic indoor environments. As demonstrated in Figure 1, these challenges manifest in three fundamental forms across both theoretical and experimental aspects [10,11,12]: (1) failure of object identification by the segmentation network; (2) incomplete segmentation results despite correct object identification; and (3) degradation of segmentation mask accuracy in boundary regions.

Unsuccessful object recognition and inadequate segmentation can cause feature points from dynamic objects to be erroneously incorporated into pose estimation, while blurred boundaries create uncertainty in the classification of feature points. The aforementioned problems impose considerable limitations on the practical performance of visual SLAM systems within complex dynamic indoor environments.

For such complex environments, it is essential to fully exploit the characteristics of the environment and apply more robust methods for SLAM system processing to address the discussed challenges [13]. Based on the assumption that dynamic object motion maintains continuity within brief time intervals, we employ temporal information to predict the potential locations where semantic information is missing and recover the missing semantic information. Under the assumption that dynamic objects maintain spatial continuity in 3D space, we explore the integration of two methodologies that reflect this fundamental property to enhance our method. Depth information reflects the distance between the camera and the detected objects, and this property is exploited to restore segmentation results in cases of incomplete segmentation. The identification of dynamic objects in dynamic SLAM resembles a classification problem, and probabilistic approaches have demonstrated excellent performance when controversies arise in classification tasks [14,15,16,17]. Thus, a probability-based approach is introduced into our SLAM system as a reliable basis for feature point classification, compensating for the inadequate precision of mask boundaries. Therefore, the aforementioned benefits motivated us to leverage temporal information, depth information, and a probability-based mechanism in our dynamic visual SLAM system, particularly to address the challenges that motion blur poses for semantic information utilization in dynamic SLAM systems. In summary, our contributions are as follows:

1. We present a semantic information compensation mechanism that predicts and recovers missing semantic information based on temporal analysis. This mechanism empowers our method to cope with failure of object identification.
2. We propose a fusion method to generate segmented depth masks that incorporate reliable depth information. In contrast to semantic masks susceptible to incomplete segmentation, our proposed masks augment the integrity of RGB-derived semantic information, thus allowing superior discrimination between dynamic objects and background.
3. We introduce a probability-based detection and elimination algorithm combined with segmented depth masks, which effectively eliminates the impacts of dynamic feature points and overcomes the unreliability of mask boundaries in the presence of motion blur.

## 2. Related Works

### 2.1. Dynamic SLAM by Fusing Semantic and Geometric Constraints

For SLAM systems operating in indoor dynamic environments, eliminating the effects of moving objects is crucial [7,18]. Methods for recognizing moving objects have long been studied [7,8,9,18,19,20,21], and various types of semantic and geometric information are used to aid SLAM systems in distinguishing dynamic objects. For instance, in DynaSLAM [7] and DynaSLAM2 [7], Mask R-CNN [22] is used to segment each frame and obtain semantic masks of moving objects. Multi-view geometry is then used with the semantic masks to identify and remove moving features. Ji et al. [23] adopted SegNet [24] as their semantic module and proposed a geometry clustering method as their geometry module. To achieve the geometry clustering method, they computed an average reprojection error for all features points against their corresponding points in 3D space. Detect-SLAM [25] utilizes Single Shot Multibox Object Detector (SSD) [26] as its detector module. Detect-SLAM exclusively detects moving objects in keyframes and updates the probability of points being in motion. In DS-SLAM [20], SegNet [24] is combined with a moving consistency check to reduce the impact of moving objects, and an optional flow pyramid is then calculated to obtain matched feature points. CFP-SLAM [8] is a dynamic scene-oriented visual SLAM system based on the object detection network YOLOv5 and a coarse-to-fine probability. Furthermore, it employs projection constraints and epipolar constraints as its multi-view geometric constraints. Blitz-SLAM [12] utilizes BlitzNet [27] to obtain semantic information and generate a global point cloud map. GGC-SLAM [28] calculates epipolar distance and uses object detection results to eliminate dynamic feature points.

In the aforementioned approaches, semantic information plays an essential role, whereas multi-view geometric information serves as a complementary cue for detecting dynamic objects. Semantic information facilitates high-level understanding of scene contents, enabling efficient object categorization and behavior prediction. Therefore, addressing the unreliability of semantic information caused by motion blur becomes particularly crucial in visual SLAM systems, as it directly impacts the system’s ability to maintain consistent object tracking.

### 2.2. Dynamic SLAM Using Depth Images

Since visual SLAM can employ RGB images and depth images for localization and mapping, the use of various types of information from depth images to identify dynamic objects and dynamic feature points has been explored for some dynamic SLAM methods.

CFP-SLAM [8] uses depth images for DBSCAN clustering. Refusion [29] registers the current RGB-D frame with respect to the pose model. After registration, Refusion computes the residual of each pixel, together with depth images, to identify dynamic parts of the scene. YOLO-SLAM [19] uses depth images and Darknet19-YOLOv3 to perform Geometric Depth RANSAC clustering. Jing et al. [30] utilized depth image sequences and semantic labels to generate a clean point cloud map with semantic information. In [23], Ji et al. segmented each depth image into N clusters using the K-means algorithm for dynamic object detection and removal. In Virgolino et al. [31], depth images are processed using a 3D Kalman filter and short-term data association mechanism to achieve dynamic object classification. Vincent et al. [32] developed an approach called DOTMask to improve both localization and mapping in visual SLAM. For DAM-SLAM [33], a depth attention module and adaptive impact factor were proposed to achieve dynamic point removal. OVD-SLAM [16] employs a chi-square test with YOLOv5 and depth images to perform foreground and background segmentation. The chi-square test is then implemented once more to identify the bounding box of dynamic objects. In YPR-SLAM [34], the Geometric Depth-PROSAC algorithm was introduced to effectively utilize depth images. Li et al. [35] developed a novel method that uses depth values to segregate non-static points. This method included an SDF-based feature point filtering algorithm.

The aforementioned methods have demonstrated promising results, yet opportunities for improvement remain. In our approach, we attempt to address the uncertain boundaries caused by motion blur by incorporating depth information.

## 3. Method

**Figure 2:**
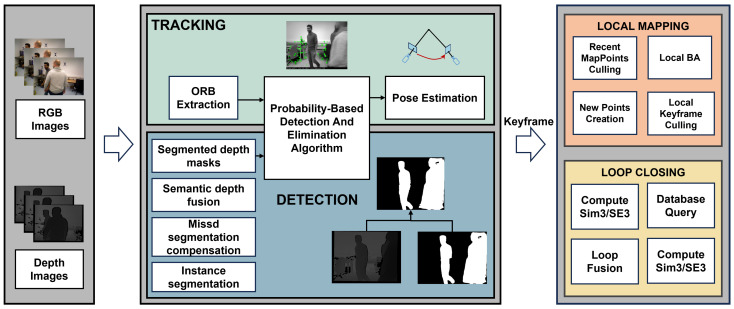
The framework of our method. A new detection thread has been added to the ORB-SLAM2 framework. In this detection thread, the RGB images are fed into a segmentation network to obtain segmented masks. Following the missed segmentation compensation process, these masks are combined with their corresponding depth images to generate segmented depth masks. The feature points and segmented depth masks are input into a probability-based detection and elimination algorithm, which decides whether to retain or discard feature points.

The overview of our SLAM system is shown in Figure 2.

Within our SLAM system architecture, a detection thread is established and coupled with the tracking thread through a probability-based detection and elimination algorithm to complete our overall framework. We present the details of our system architecture in this section. The ORB feature is employed for the overall system. Our loop closing module follows the same approach as ORB-SLAM2, while in the mapping stage, our system constructs a global dense 3D point cloud map. Initially, the missed segmentation compensation mechanism is employed to address the segmentation deficiencies in our methodology. Subsequently, a fusion method combining depth information with semantic information extracted from RGB images is utilized. Finally, the details of the probability-based detection and elimination algorithm are demonstrated.

### 3.1. Missed Segmentation Compensation

Semantic information was primarily obtained from the instance segmentation network SparseInst [36]. Within the segmentation output, humans were classified as dynamic objects, where the bounding box information for each dynamic object was extracted and stored, denoted as $B=\left\{B^{i}\right\},i=1,2,\cdots ,o$. $B^{i}$ represents an individual bounding box, where *o* represents the total number of bounding boxes. Subsequently, the segmentation results were converted into masks through binarization processing, in which dynamic object regions are represented in white, while the remaining areas are displayed in black. However, the missed segmentation problem arises due to the blurred boundaries of moving objects in images. This renders our method incapable of handling dynamic objects. Furthermore, the unexpected introduction of dynamic objects leads to the acquisition of extensive erroneous keypoints within our SLAM system, with subsequent induction of inter-frame correspondence anomalies, thus compromising overall system performance. Therefore, recovering the semantic information in frames with missed segmentation is crucial. When the missed segmentation problem occurs, the mask obtained from the current frame shows significant differences compared to the masks from previous frames. Specifically, we choose to represent this difference using a ratio *r*:

(1) $$r=\frac{A_{c}}{A_{c-1}}$$

where $A_{c}$ denotes the area of the white region in the mask corresponding to the current frame and $A_{c-1}$ denotes the area of the white region in the mask corresponding to the last frame. Through empirical evaluations, we establish the criterion that a missed segmentation problem arises when r<0.9. Local template matching was then utilized to locate objects with missed segmentation, after which the prediction process was initiated. In our prediction process, the coordinates of the upper left corner of the boundary box $B^{i}$ are saved. These coordinates are integrated with the timestamp corresponding to the frame in which they are located. This combination is treated as a 3D point $Q^{i}$, denoted as

(2) $$Q^{i}=\left(x_{i},y_{i},t_{i}\right)$$

where $x_{i}$ is the horizontal coordinate, $y_{i}$ is the vertical coordinate, and $t_{i}$ is the timestamp. Using the abovementioned combination, the prediction problem is transformed into a line fitting task for a series of points $Q^{i}$ in the 3D space. We employ the least squares technique to fit these points. Specifically, the equation of a three-dimensional line can be expressed as follows:

(3) $$\frac{x-x_{0}}{m}=\frac{y-y_{0}}{n}=\frac{t-t_{0}}{p}$$

where $\left(x_{0},y_{0},t_{0}\right)$ represents a point on this line and m,n,p are constant values. This line can also be expressed as

(4) $$x=\frac{m}{p}t+\left(x_{0}-\frac{m}{p}t_{0}\right)=k_{1}t+b_{1},y=\frac{n}{p}t+\left(y_{0}-\frac{n}{p}t_{0}\right)=k_{2}t+b_{2}$$

Using the L2 norm of the residuals, the parameters $k_{1}$, $b_{1}$, $k_{2}$ and $b_{2}$ can be estimated as follows:

(5) $$\hat{k_{1}},\hat{k_{2}},\hat{b_{1}},\hat{b_{2}}=\underset{k_{1},b_{1}}{arg min}\;{∥x_{i}-k_{1}t_{i}-b_{1}∥}_{2}^{2}+\underset{k_{2},b_{2}}{arg min}\;{∥y_{i}-k_{2}t_{i}-b_{2}∥}_{2}^{2}$$

Utilizing the aforementioned formula, the values for the parameters $\hat{k_{1}}$, $\hat{b_{1}}$, $\hat{k_{2}}$ and $\hat{b_{1}}$ are estimated as follows:

(6) $$\hat{k_{1}}=\frac{n\sum_{i=1}^{n}x_{i}t_{i}-\sum_{i=1}^{n}x_{i}\times \sum_{i=1}^{n}t_{i}}{n\sum_{i=1}^{n}t_{i}^{2}-\sum_{i=1}^{n}t_{i}\times \sum_{i=1}^{n}t_{i}},\hat{b_{1}}=\frac{\sum_{i=1}^{n}x_{i}-k_{1}\sum_{i=1}^{n}t_{i}}{n}$$

(7) $$\hat{k_{2}}=\frac{n\sum_{i=1}^{n}y_{i}t_{i}-\sum_{i=1}^{n}y_{i}\times \sum_{i=1}^{n}t_{i}}{n\sum_{i=1}^{n}t_{i}^{2}-\sum_{i=1}^{n}t_{i}\times \sum_{i=1}^{n}t_{i}},\hat{b_{2}}=\frac{\sum_{i=1}^{n}y_{i}-k_{2}\sum_{i=1}^{n}t_{i}}{n}$$

where *n* denotes the number of 3D points $Q^{i}$ involved in the estimation process. In the proposed method, 3D points $Q^{i}$ from the three preceding frames of the current frame are selected for estimation, hence n=3. For the current frame where missed segmentation occurs, the timestamp of this frame is already known. Consequently, the predicted 3D points for the current frame are as follows:

(8) $$\left(x_{p},y_{p},t_{c}\right)=\left(\hat{k_{1}}t_{c}+\hat{b_{1}},\hat{k_{2}}t_{c}+\hat{b_{2}},t_{c}\right)$$

where $x_{p}$, $y_{p}$ are the predicted horizontal coordinate and the predicted vertical coordinate, and $t_{c}$ is the timestamp of current frame. After predicting the potential location for the bounding box of the missing mask within the current frame, we copy the mask from the previous frame to this location, thereby achieving the recovery of missing semantic information.

### 3.2. Fusion of Depth Information with RGB-Derived Semantics

**Figure 3:**
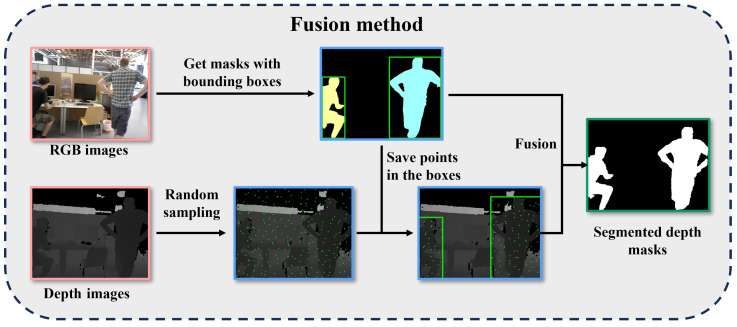
The fusion method process. The images in orange boxes are the inputs, the images in blue boxes are the visualization of intermediate steps, and the image in the green box is the output.

The fusion method process is presented in Figure 3. As previously described, semantic information was recovered through implementation of the missed segmentation compensation mechanism.

The first step involves sampling from depth images. We perform stratified, rather than random, sampling directly on the depth images. Stratified sampling can reduce sampling error and bias, thereby accurately reflecting the characteristics of the population. We divide depth image $I_{D}$ into *n* regions, denoted as $R_{n}$. Each region has uniform length and width.

(9) $$I_{D}=\left\{R_{1},R_{2},\ldots ,R_{n}\right\}$$

We define a vector $p_{l}\in R^{3}$ to represent the characteristics of a sampling point. This vector can be expressed as

(10) $$p_{l}=\left(p_{lg},p_{lc}\right)$$

where $p_{lg}$ is the grayscale value of $p_{l}$ and $p_{lc}$ is the coordinates of $p_{l}$. Given each $R_{n}$, $p_{l}$ can be expressed as

(11) $$p_{l}∼U\left(R_{n}\right)$$

where $U(R_{n})$ is uniform sampling function. The probability density function f of uniform sampling function $U(R_{n})$ can be written as follows:

(12) $$f\left(p_{lc}\right)=\left\{\begin{array}{cc} \frac{1}{G} & ,\;\mathrm{if}\;p_{lc}\in R_{n}\\ 0 & ,\;\mathrm{otherwise} \end{array}\right.$$

where *G* is the area of $R_{n}$. Let $P_{rs}$ be the set of $p_{l}$. $P_{rs}$ can be expressed using the following equation:

(13) $$P_{rs}=\left\{p_{1},p_{2},\ldots ,p_{l}\right\}$$

We apply a filtering process to refine $P_{rs}$, which is based on the bounding boxes B. For each bounding box $B^{i}$, the filtering process can be formulated as follows:

(14) $$p_{f}^{i}=p_{l},if\;p_{lc}\;is\;within\;B^{i}$$

where $p_{f}^{i}\in R^{3}$ is the outcome of filtering process applied to $p_{l}$. Therefore, $p_{f}^{i}$ has the same representation as $p_{l}$ and can be expressed as

(15) $$p_{f}^{i}=\left(p_{fg}^{i},p_{fc}^{i}\right)$$

where $p_{fg}^{i}$ is the grayscale value of $p_{f}^{i}$, and $p_{fc}^{i}$ represents the coordinates of $p_{f}^{i}$. Let $P_{f}^{i}$ be the set of $p_{f}^{i}$, and $P_{f}^{i}$ can be denoted as

(16) $$P_{f}^{i}=\left\{p_{1}^{i},p_{2}^{i},\ldots ,p_{f}^{i}\right\}$$

To speed up our fusion method, we do not perform random sampling in the bounding boxes. We use the bounding boxes $B^{i}$ and the sampled points $P_{f}^{i}$ to extract human regions within the depth images. Our approach is based on the property that grayscale values of a continuous object maintain spatial continuity within depth images. Therefore, for every bounding box $B^{i}$ in depth images, the average grayscale values $g_{h}^{i}$ can be computed as follows:

(17) $$g_{h}^{i}=\frac{\sum_{f=1}^{n}p_{fg}^{i}}{n}$$

where *n* is the total number of $p_{fg}^{i}$. This computed value $g_{h}^{i}$, denoted as “human average value”, is regard as the average grayscale value of the human region $H^{i}$ corresponding to the bounding box $B^{i}$. Given “human average value” $g_{h}^{i}$, a range called “human value range” $O_{h}^{i}$ can be written as

(18) $$O_{h}^{i}=\left[g_{h}^{i}-\alpha ,g_{h}^{i}+\alpha \right]$$

where α is the threshold used to determine the upper and lower limits of this range. We analyzed the distribution of grayscale values within the bounding box $B^{i}$ and retained those within three standard deviations. If a grayscale value is excluded from the random sampling process but within the range $O_{h}^{i}$, we add this value into $O_{h}^{i}$ and subsequently update $O_{h}^{i}$. Grayscale values within $O_{h}^{i}$ are regarded as part of the overall grayscale value of this human region. In this way, we can effectively avoid the problem of missed samples that may occur in the sampling process. For each grayscale value belonging to “human value range” $O_{h}^{i}$, we extract the regions $M_{d}^{i}$ corresponding to this grayscale value in the bounding box $B^{i}$ and then copy this region to the semantic masks $M_{s}$. The corresponding regions are set as white in the masks. Using these approaches, we create segmented depth masks $M_{sd}$ containing both depth and semantic information.

(19) $$M_{sd}=M_{s}\cup \sum M_{d}^{i}$$

We apply a dilation function to the segmented depth masks. In the dilation function, the kernel size is a critical parameter that affects the performance of the algorithm, and kernel selection will be discussed in the experimental section.

Finally, segmented depth masks are fed into a probability-based detection and elimination algorithm, which will be detailed in the subsequent section.

### 3.3. The Probability-Based Detection and Elimination Algorithm

A probability-based detection and elimination algorithm is developed to identify and eliminate dynamic feature points, in which “probability” refers to the likelihood that a feature point belongs to a dynamic object.

**Figure 4:**
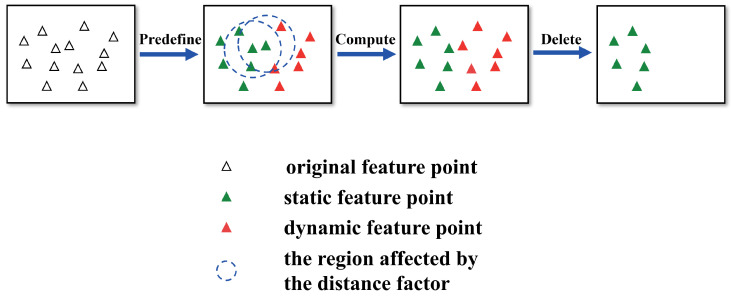
The process of probability-based detection and elimination algorithm.

For the ORB feature points extracted in each frame, the entire approach of this algorithm is illustrated in Figure 4.

The first step is to “Pre-define”, which means to coarsely assign a probability of being a dynamic point to all ORB feature points. Relying on segmented depth masks $M_{sd}$, if a feature point $x_{i}$ with i=1,2,3,⋯,m falls within the white region of segmented depth masks, we consider this point a dynamic feature point $x_{i}^{d}$ with probability set to 100%; otherwise, it is a static point $x_{i}^{s}$ with probability set to 0%, as defined in Equation (20).

(20) $$P\left(x_{i}\right)=\left\{\begin{array}{cc} 100\% & ,\;\mathrm{if}\;x_{i}\in M_{sd}\\ 0\% & ,\;\mathrm{if}\;x_{i}\notin M_{sd} \end{array}\right.$$

The second step is to “Compute”, which is to refine the probability of the detected points.

For each static feature point $x_{i}^{s}$, all neighbor feature points with a Euclidean distance of less than *D* to $x_{i}^{s}$ are searched using KD tree and recorded into a set $X_{j}=\left\{x_{k}\right\}$, with k=1,2,3,…,n. The refined probability $P(x_{i}^{s})$ is computed as follows:

(21) $$P\left(x_{i}^{s}\right)=\left\{\begin{array}{cc} \sum_{k=1}^{n}\frac{\lambda \left(r_{k}\right)P\left(x_{k}\right)}{n} & ,\;\mathrm{if}\;n\;>0\\ 0 & ,\;\mathrm{otherwise} \end{array}\right.$$

where $\lambda (r_{k})$ is a distance decay factor. $r_{k}\left(r_{k}\leq D\right)$ is the Euclidean distance between $x_{i}^{s}$ and $x_{k}$. The distance decay factor is defined as follows:

(22) $$\lambda \left(r_{k}\right)=\;c_{1}e^{-c_{2}r_{k}}$$

Here, $c_{1}$, $c_{2}$ are the scaling and exponential parameters of the distance decay factor. The natural exponential function is used to reflect the effect of Euclidean distance. If n=0, this indicates that there are no neighbor feature points around $x_{i}^{s}$ that can be utilized to refine $P(x_{i}^{s})$, then $P(x_{i}^{s})$ is set to 0%. By using the aforementioned method, we obtain the fine probability of these original static points.

Therefore, dynamic feature points do not only exist within mask boundaries and sometimes also appear outside the mask boundaries. The “Compute” step effectively extends the possible locations of dynamic feature points.

The last step is to “Delete”. The “Delete” step is designed to mitigate the adverse effects of dynamic feature points. In the “Delete” step, a filtering mechanism is employed to eliminate all the dynamic feature points that we have identified. We set a threshold θ for the classification of dynamic feature points. Through this process, we separate all feature points into two categories, $x_{i}^{d}$ and $x_{i}^{s}$. $x_{i}^{d}$ means feature point with its probability $P(x_{i}^{d})>\theta$ and $x_{i}^{s}$ means a feature point of probability $P(x_{i}^{s})<\theta$. If the fine probability of some original static points exceeds θ, they will be classified as dynamic feature points. This demonstrates that despite being located outside mask boundaries, these feature points are computationally identified as being unreliable due to the influence of motion blur. Using the filtering mechanism, all dynamic points are located and the eliminated during subsequent processing stages. This mechanism prevents incorrect data associations caused by dynamic feature points. Finally, static feature points, which are inherently more reliable, are utilized for the pose estimation task within the SLAM system. The other modules of the SLAM system also use static feature points instead of the original extracted feature points.

## 4. Experimental Results and Discussion

In this study, five “fr3” sequences from the TUM RGB-D dataset and five sequences from the Bonn dataset were selected for the evaluation of our proposed method [29,37]. The five “fr3” sequences from the TUM RGB-D dataset contain both high- and low-dynamic environment sequences, in which the high-dynamic environment sequences, named “fr3/walking” (fr3/w for short), contain four kinds of camera motion: xyz, halfsphere, static, rpy. The low-dynamic environment sequences are called “fr3/sitting/static” (fr3/s/static for short). From the Bonn dataset, we selected sequences of “crowd”, with multiple people present, as well as “person_tracking” (person for short), which are sequences focused on person tracking. Absolute trajectory error (ATE) was employed to evaluate the differences between the estimated trajectories and the ground truth trajectories. Root mean square error (RMSE) and standard deviation (S.D.) were employed for the statistical analysis of ATE, as the evaluation metrics of different SLAM methods. With the transformation matrix $S_{t}$ corresponding to the estimated camera pose $P_{t}$ and ground truth $G_{t}$ at frame *t* using the least squares method, ATE and the corresponding RMSE and S.D. can be computed as

(23) $$ATE=G_{t}^{-1}SP_{t}$$

(24) $$RMSE_{ATE}=\sqrt{\frac{1}{n}\sum_{i=1}^{n}ATE_{i}^{2}}$$

(25) $$S.D_{.ATE}=\sqrt{\frac{1}{n}\sum_{i=1}^{n}{\left(ATE_{i}-\bar{ATE}\right)}^{2}}$$

where *n* was the number of frames in a sequence.

Initially, we showed the effectiveness of our missed segmentation compensation and fusion method. Subsequently, we compared our method with different baseline methods. We then performed ablation experiments to verify the effect of each module in our method. After that, we presented the dense mapping results and then analyzed and visualized the influence of mask size on performance. Lastly, we illustrated the runtime efficiency of our method. The hyperparameters utilized in our methodology were configured according to the default settings prescribed by ORB-SLAM2, except that the number of ORB features to be extracted from each image had been increased to 3000 from the default 1000. We ran each sequence five times to minimize systematic errors. All experiments were conducted on a computer with Intel i5-13400F CPU, 16 GB memory, and NVIDIA GeForce GTX 1660 SUPER GPU.

### 4.1. Evaluation of the Missed Segmentation Compensation and Fusion Method

**Figure 5:**
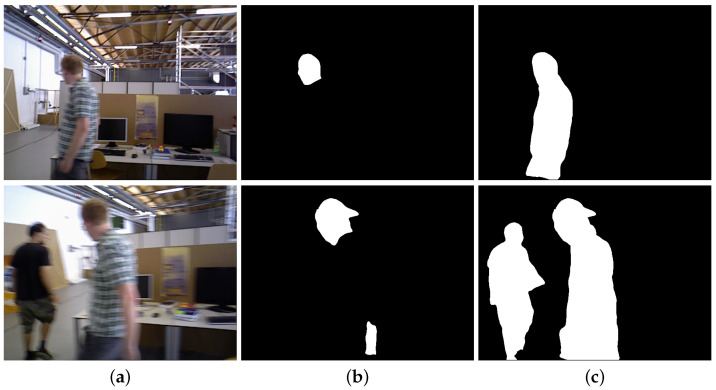
Illustration of the effect of our proposed missed segmentation compensation mechanism: (**a**) the original RGB images; (**b**) semantic masks before missed segmentation compensation; (**c**) our missed segmentation compensation results.

**Figure 6:**
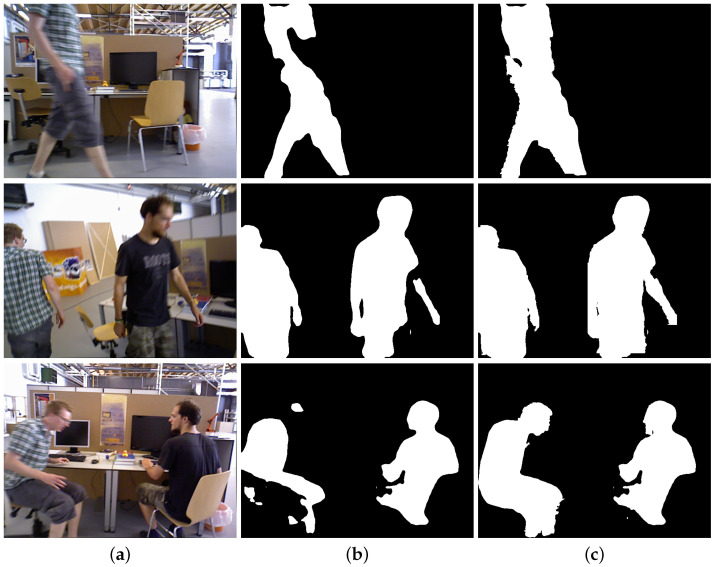
Illustration of the effect of our proposed depth fusion method in completing the semantic masks in segmentation: (**a**) the original RGB images; (**b**) semantic masks without fusing depth information; (**c**) our segmented depth masks.

In dynamic visual SLAM scenarios, the motion of objects and the camera poses significant challenges to segmentation networks, resulting in problems of missed and incomplete segmentation. Semantic masks that suddenly disappear or are partially missing compromise the effectiveness of our method. Consequently, we predict the position and supplement the semantic masks for frames that have experienced missed segmentation, utilizing depth information to complement the partially segmented masks. Figure 5 illustrates cases of missed segmentation problems found in two different sequences along with the results when using our method for compensation. Due to the non-negligible motion of dynamic objects between consecutive frames, we refrained from simply copying the correct semantic masks to the frames where segmentation failure was detected. Instead, our missed segmentation compensation mechanism effectively forecasted the potential locations of the missing semantic masks based on the results of line fitting and successfully compensated for the missing semantic masks. Figure 6 demonstrates the effectiveness of our fusion method. For the RGB images shown in the figure, although the segmentation network produced incomplete masks due to motion blur, our fusion method successfully completed these masks by leveraging depth information. The experimental results substantiate that our missed segmentation compensation mechanism and fusion method effectively recover the missing parts of human segmentation, leading to more complete and accurate dynamic object detection while maintaining robust performance.

### 4.2. Comparison with Baseline Methods

Our method was compared with five baseline methods, ORBSLAM2 [38] and four advanced dynamic SLAM methods, Dyna-SLAM [7], Crowd-SLAM [39], YOLO-SLAM [19], and SG-SLAM [40]. The baseline data are from the source literature, and “/” means that the corresponding data were not provided in their source literature.

**Figure 7:**
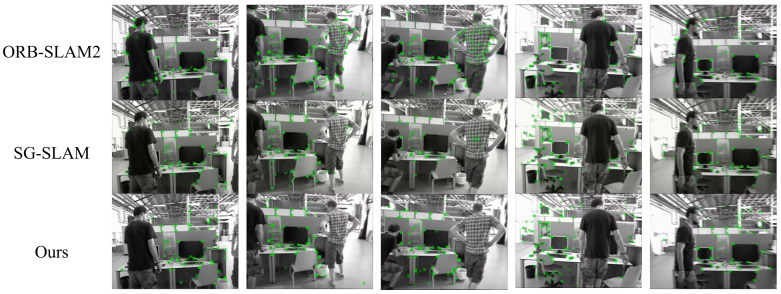
Keypoint filtering results compared with ORB-SLAM2 and SG-SLAM.

**TUM RGB-D dataset**. Our quantitative results are displayed in Table 1. The keypoint filtering results compared with ORB-SLAM2 and SG-SLAM are shown in Figure 7.

The experimental results demonstrate that our method outperforms the baseline methods in terms of ATE in both high- and low-dynamic environments. In high-dynamic sequences, our method achieved an average reduction of 96.5% in RMSE and 96.6% in S.D. compared to ORBSLAM2. Moreover, in the “fr3/w/half” sequence characterized by severe motion blur, our method demonstrated optimal performance, with RMSE reduced by 94.5% and S.D. reduced by 95.5% compared to ORBSLAM2, and improvements of 14.8% in RMSE and 15.4% in S.D. over SG-SLAM. For the “fr3/w/rpy” sequence, both SG-SLAM and our method exhibited superior performance, whereas YOLO-SLAM did not yield comparable results. Experimental results from the “fr3/w/rpy” sequence demonstrate that degradation in the performance of YOLO-SLAM is primarily attributed to its substantial dependence on object detection outputs, wherein the detection network exhibits significant accuracy deterioration during the 45-degree camera rotation, thereby adversely affecting the system’s overall functionality. In low-dynamic environments, all baseline methods showed minimal improvements over ORBSLAM2, whose performance is already promising due to the limited motion of both camera and objects in these sequences. The visualization results showed that compared to ORB-SLAM2, both our method and SG-SLAM effectively filtered out dynamic feature points associated with moving humans, leading to more accurate localization of the SLAM system. Furthermore, while SG-SLAM retained feature points at human boundaries, our method demonstrated enhanced robustness in scenarios with frequent human movement by more precisely identifying and filtering these boundary features, effectively reducing localization drift.

**Figure 8:**
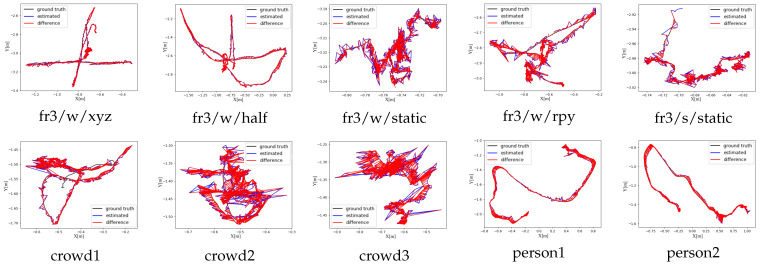
The ATE [m] results of our method on the TUM RGB-D and Bonn datasets. The results on the TUM dataset are presented in the first row. The results on the Bonn dataset are presented in the second row.

We draw our trajectory as plots using the evaluation tool provided with the TUM dataset. These ATE plots are shown in the first row of Figure 8.

**Bonn dataset**. The tracking results are exhibited in Table 2.

Compared with the TUM RGB-D dataset, which serves as a standard benchmark for SLAM evaluation, the Bonn dataset exhibits more complex dynamic scenarios, involving the motion of multiple objects, and includes more frequent occlusions and complex object interactions, which must be accurately handled by the SLAM system. Our method demonstrated a significant improvement over ORB-SLAM2, with superior robustness in handling dynamic objects and maintaining accurate camera pose estimation. Specifically, when evaluated on Bonn dataset sequences, our approach achieved average reductions of 96.6% in RMSE and 96.7% in S.D. compared to ORBSLAM2. Furthermore, when compared with the state-of-the-art SG-SLAM system, our method performed competitively, achieving comparable or better results using these sequences. Quantitative analysis revealed that our approach achieved a 24.8% reduction in RMSE compared to SG-SLAM, while demonstrating an even more significant improvement of 35.8% in S.D. The experimental results validate the effectiveness of our proposed method and its ability to handle complex dynamic environments. The ATE plots are shown in the second row of Figure 8.

### 4.3. Ablation Experiments

Ablation experiments were performed to evaluate the influence of each module in our method. “Ours” refers to our method. “w/o compensation” denotes that the missed segmentation compensation mechanism has not been applied. “w/o depth” indicates results without fusing depth information and masks provided by the segmentation network. “w/o probability” means the probability algorithm is not applied. The experimental results are shown in Table 3.

Our experimental results on the “fr3/s/static” sequence demonstrate that optimal performance is achieved when operating without the fusion of depth information and masks. This phenomenon occurs because our approach is based on an implicit assumption: in high-dynamic scenes, segmentation networks are more susceptible to motion blur than depth information, making the application of depth information more robust compared to segmentation masks. Based on this assumption, the method of using robust depth information to complete segmentation masks proved effective in sequences with dramatic camera or human motion. However, in low-dynamic scenes, this assumption became invalid: the segmentation networks were capable of producing high-quality masks, whereas the inherent inaccuracy of depth information near object boundaries introduced undesired noise into our fusion approach.

**Figure 9:**
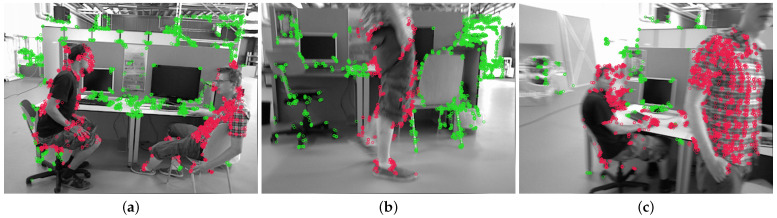
Visualization of results for our probability-based detection and elimination algorithm. The results from three different sequences are each presented in (**a**–**c**). In the visualization results, feature points that are ultimately determined to be static are represented in green, while those classified as dynamic are depicted in red.

We visualized our probability-based detection and elimination algorithm in different sequences, as illustrated in Figure 9. In Figure 9, three sequences are presented, where feature points are colored based on their refined classification: green for static feature points and red for dynamic feature points. These dynamic feature points are mainly situated near dynamic objects. The green feature points represent stable environmental features that are crucial for accurate pose estimation, while the red feature points effectively identify regions of dynamic activity that require special handling in the SLAM pipeline. Utilizing our probability algorithm, we implemented adaptive propagation of dynamic points around object boundaries, successfully addressing the precision limitations of binary classification methods that rely exclusively on mask boundaries. We judged these dynamic feature points to be unreliable, and they were disqualified from participation in the subsequent threads of the SLAM process. Ablation experiments show that the probability-based detection and elimination algorithm enhances the performance of our method, particularly in sequences like “fr3/w/rpy” and “fr3/w/half”, which exhibit significant motion blur caused by object and camera motions. In summary, our method achieved superior performance, highlighting that the three modules were successfully integrated.

### 4.4. Dense 3D Mapping

**Figure 10:**
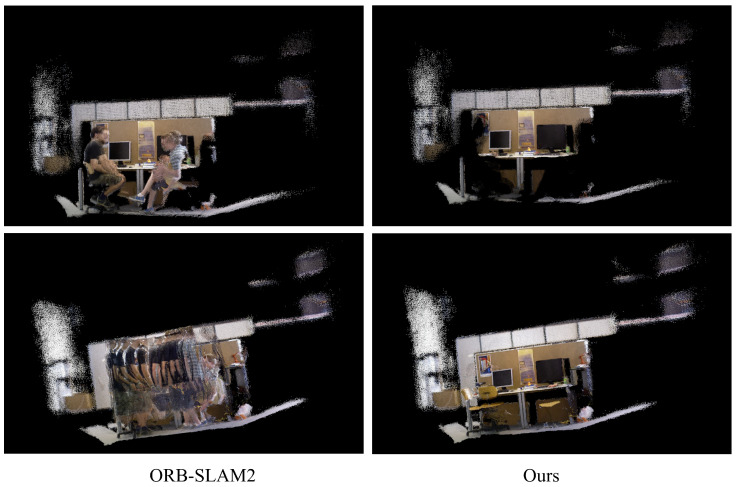
Dense 3D maps for the TUM RGB-D dataset. The sequence of the top line is “fr3/s/static”, and the sequence of the bottom line is “fr3/w/static”.

In this section, we evaluated our dense 3D mapping results using the TUM RGB-D dataset. The comparison of mapping results between our method and ORB-SLAM2 is presented in Figure 10. The dense map generated by ORB-SLAM2 exhibited severe ghost effects in the presence of dynamic human motion. In contrast, our approach demonstrated superior robustness against dynamic objects, exhibiting significantly reduced ghosting effects in the dense mapping process even in scenarios where motion blur was introduced by human movement. Overall, our method consistently produced clean and coherent dense 3D maps of the environment.

### 4.5. Impact of Dilation Kernel Size on Performance

**Figure 11:**
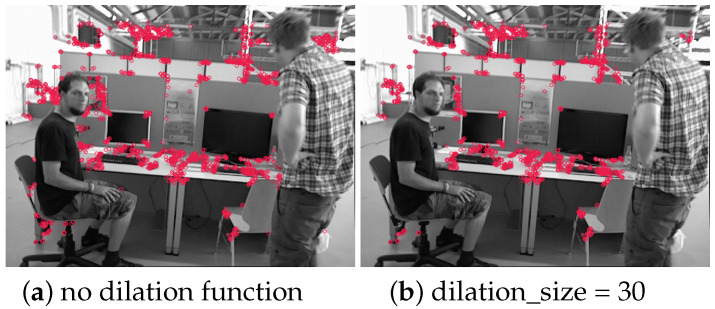
Visualization of the number of feature points under different kernel sizes: ORBSLAM2 (**left**), our method without using dilation function (**middle**), and using dilation function with dilation_size = 30 (**right**).

The masks were processed using the dilation function. The parameter “dilation_size” was set when constructing the kernel that is used in the dilation function. Visualizations are depicted in Figure 11.

**Figure 12:**
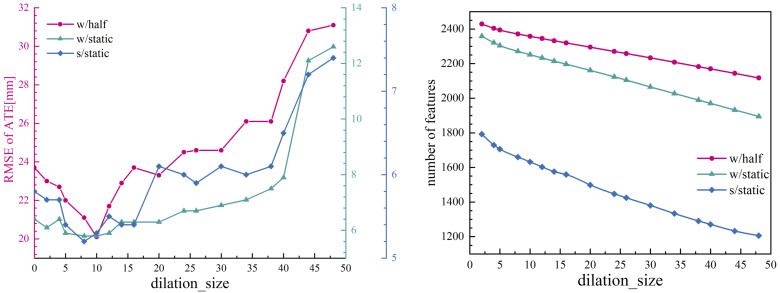
Impacts of different dilation_size for our proposed SLAM system. Plots of SLAM performance under different dilation_size (**left**), and the average number of feature points used by the SLAM system under different dilation_size (**right**).

The experimental results and the average number of feature points used per image under different kernel sizes are presented in Figure 12.

The empirical findings indicate that different kernel sizes do not cause a sudden changes in the average number of feature points utilized. However, it is observed that the performance of the SLAM system achieves a local optimal outcome when a certain kernel size is applied. The use of oversized masks decreases the quantity of static points incorporated into the SLAM system, consequently leading to a discernible decrease in system performance. Conversely, the utilization of undersized masks introduces dynamic points into the SLAM system, similarly leading to a degradation in overall performance. These findings suggest that kernel size plays a critical role in balancing the inclusion of static and dynamic feature points, thereby influencing the efficacy of the SLAM system. In practice, we found “dilation_size=10” was the optimal choice.

### 4.6. Runtime Analysis

To complete the evaluation of our method, we considered the average computation time, as illustrated in Table 4. During empirical operation, we discovered that we could not run our system using the original parameters set by ORBSLAM2, so we adjusted “nFeature” to 3000. “Others” denotes the remaining system modules, which include loop closing and local mapping. The sequence “fr3/w/half” is more intricate compared to other sequences, requiring extensive computational resources for the SLAM system to utilize loop closing and local mapping in order to achieve accurate trajectory estimation, consequently entailing a longer runtime. In the “fr3/w/static” sequence, the human bodies are always present in the frame, leading to a larger computational requirement for Fusion method. SparseInst achieved an average segmentation speed of 17.1 ms per image. According to the runtime analysis, our method is capable of fulfilling real-time demands.

## 5. Conclusions

In this paper, we present our work in addressing the challenges of acquiring reliable semantic information in dynamic SLAM applications when there is motion blur present. We developed multiple strategies to minimize the influence of motion blur on visual SLAM system performance, as follows: first, a missed segmentation compensation mechanism using temporal information was developed to address the missed detection of dynamic objects; second, a fusion method that combines depth information with the semantic information extracted from RGB images was proposed to improve the accuracy in distinguishing between dynamic objects and the background; third, a probability-based detection and elimination algorithm was introduced to improve the results for the detection of dynamic feature points located at the boundaries of dynamic objects. Based on the experimental results, we can conclude the following: first, using temporal information to predict the motion and fusing depth information, it is possible to improve the robustness of segmentation to address motion blur problems in dynamic scenes; second, probability-based mechanisms can be used to effectively remove unreliable points for localization. In summary, our proposed multi-strategy visual SLAM system is effective in dealing with localization issues in dynamic indoor environments.

Although good results can be achieved using our method, our study only considered the impact of humans in the environment. Further research is required to expand the adaptability of our method. Moreover, the experimental results indicate that the trajectories estimated using our method and other similar approaches still exhibit certain deviations from the ground truth. This problem may arise from the insufficient robustness of the ORB feature points against rotational motion. In addition, research efforts on the substitution of ORB features with deep learning networks are actively being pursued [41]. This represents a possible avenue for achieving more accurate localization using dynamic SLAM systems. Therefore, in our future work, we plan to broaden the detection range of moving objects, particularly those affected by human intervention, and investigate the feasibility of using other feature points to enhance the robustness of our system.

## Figures and Tables

**Table 1:** Results of metrics for absolute trajectory error (ATE) [m] using the TUM RGB-D dataset. The best results are in bold.

Sequences	ORBSLAM2		DynaSLAM		Crowd-SLAM		YOLO-SLAM		SG-SLAM		Ours	
	**RMSE**	**S.D.**	**RMSE**	**S.D.**	**RMSE**	**S.D.**	**RMSE**	**S.D.**	**RMSE**	**S.D.**	**RMSE**	**S.D.**
fr3/w/xyz	0.729	0.386	0.016	0.009	0.020	0.017	0.015	**0.007**	0.015	0.008	**0.014**	**0.007**
fr3/w/half	0.419	0.245	0.030	0.016	0.026	0.022	0.028	0.014	0.027	0.013	**0.023**	**0.011**
fr3/w/static	0.367	0.161	0.007	**0.003**	0.007	0.007	0.007	0.004	0.007	**0.003**	**0.006**	**0.003**
fr3/w/rpy	0.775	0.411	0.035	0.019	0.044	0.031	0.216	0.100	0.032	0.019	**0.031**	**0.018**
fr3/s/static	0.009	0.004	/	/	0.008	0.007	0.007	**0.003**	0.006	**0.003**	**0.005**	**0.003**

**Table 2:** Results of metrics for absolute trajectory error (ATE) [m] using the Bonn dataset.The best results are in bold.

Sequences	ORBSLAM2		DynaSLAM		Crowd-SLAM		YOLO-SLAM		SG-SLAM		Ours	
	**RMSE**	**S.D.**	**RMSE**	**S.D.**	**RMSE**	**S.D.**	**RMSE**	**S.D.**	**RMSE**	**S.D.**	**RMSE**	**S.D.**
crowd1	0.533	0.361	**0.016**	/	0.018	/	0.033	/	0.023	0.014	**0.016**	**0.008**
crowd2	1.311	0.257	0.031	/	**0.030**	/	0.423	/	0.060	0.040	0.031	**0.018**
crowd3	0.780	0.306	0.038	/	0.034	/	0.069	/	0.032	0.022	**0.026**	**0.017**
person1	0.796	0.709	/	/	/	/	0.157	/	0.040	0.014	**0.038**	**0.012**
person2	0.898	0.405	/	/	/	/	0.037	/	0.038	0.016	**0.034**	**0.013**

**Table 3:** Results of metrics for absolute trajectory error (ATE) [m] with different modules.The best results are in bold.

Sequences	Ours		w/o Compensation		w/o Depth		w/o Probability	
	**RMSE**	**S.D.**	**RMSE**	**S.D.**	**RMSE**	**S.D.**	**RMSE**	**S.D.**
fr3/w/xyz	**0.0143**	**0.0072**	0.0150	0.0075	0.0153	0.0075	0.0156	0.0077
fr3/w/half	**0.0233**	**0.0114**	0.0245	0.0128	0.0244	0.0121	0.0253	0.0134
fr3/w/static	**0.0058**	**0.0027**	0.0064	0.0029	0.0065	0.0030	0.0064	0.0032
fr3/w/rpy	**0.0314**	**0.0177**	0.0331	0.0196	0.0329	0.0180	0.0344	0.0211
fr3/s/static	0.0054	0.0026	0.0060	0.0029	**0.0053**	**0.0025**	0.0064	0.0028

**Table 4:** Average computation time [ms].

Sequences	ORB Extraction	Compensation	Fusion	Probability	Others	Total
fr3/w/half	14.44	4.35	7.34	4.57	16.79	47.49
fr3/w/static	14.61	4.14	7.58	4.56	8.58	39.47
fr3/s/static	15.14	4.37	11.85	4.29	7.84	43.49

## Data Availability

The dataset used in this paper is the public TUM dataset and Bonn dataset. The download addresses are as follows: TUM dataset at https://vision.in.tum.de/data/datasets/rgbd-dataset (accessed on 7 January 2025), Bonn dataset at https://www.ipb.uni-bonn.de/data/rgbd-dynamic-dataset/index.html (accessed on 7 January 2025).
